# KCN Chemical Etching of van der Waals Sb_2_Se_3_ Thin Films Synthesized at Low Temperature Leads to Inverted Surface Polarity and Improved Solar Cell Efficiency

**DOI:** 10.1021/acsaem.3c01584

**Published:** 2024-01-22

**Authors:** Maykel Jiménez-Guerra, Lorenzo Calvo-Barrio, Jose Miguel Asensi, Ivan Caño-Prades, Shunya Yan, Esther Barrena, Joaquim Puigdollers, Zacharie Jehl, Yudania Sánchez, Edgardo Saucedo

**Affiliations:** † Electronic Engineering Department, Universitat Politècnica de Catalunya (UPC), Photovoltaic Lab − Micro and Nano Technologies Group (MNT), EEBE, Av Eduard Maristany 10-14, Barcelona 08019, Catalonia, Spain; ‡ Barcelona Center for Multiscale Science & Engineering, Universitat Politècnica de Catalunya (UPC), Av Eduard Maristany 10-14, Barcelona 08019, Catalonia, Spain; § Centres Científics i Tecnològics (CCiTUB), Universitat de Barcelona, C. Lluis Solé i Sabaris 1-3, 08028 Barcelona, Spain; ∥ IN2UB, Departament d′Enginyeria Electrònica i Biomèdica, Universitat de Barcelona, C. Martí i Franquès, 1, 08028 Barcelona, Spain; ⊥ Departament de Física Aplicada, Universitat de Barcelona, C. Martí i Franquès, 1, 08028 Barcelona, Spain; # Institut de Ciència de Materials de Barcelona (ICMAB), Carrer dels Til·lers, Bellaterra 08193, Spain; ∇ Institut de Recerca en Energia de Catalunya (IREC), Jardins de les Dones de Negre, 1, 08930 Sant Adrià del Besòs, Spain

**Keywords:** Antimony selenide, Etching, Thin film, Buried junction, KCN etching, Bromine etching

## Abstract

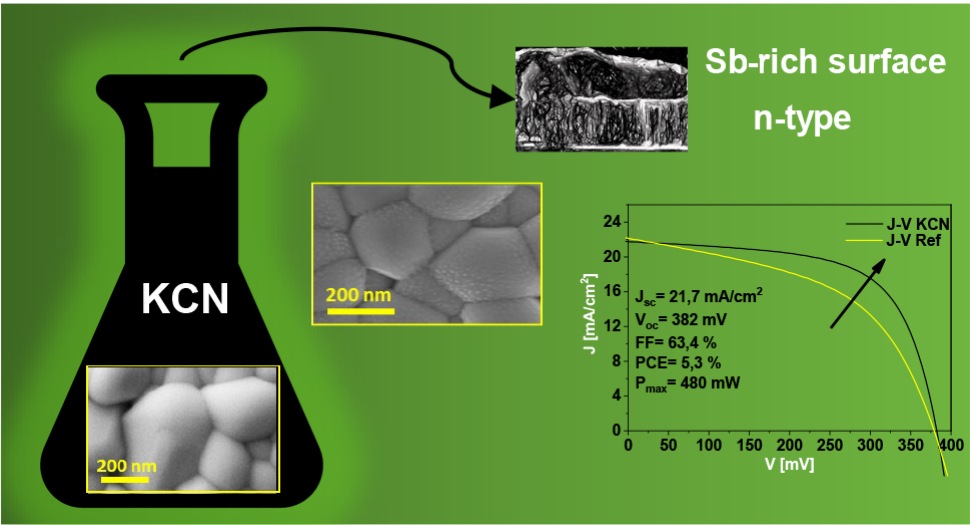

Recent developments in Sb_2_Se_3_ van der Waals material as an absorber candidate for thin film photovoltaic applications have demonstrated the importance of surface management for improving the conversion efficiency of this technology. Sb_2_Se_3_ thin films’ versatility in delivering good efficiencies in both superstrate and substrate configurations, coupled with a compatibility with various low-temperature deposition techniques (below 500 °C and often below 350 °C), makes them highly attractive for advanced photovoltaic applications. This study presents a comparative analysis of the most effective chemical etchings developed for related thin film chalcogenide technologies to identify and understand the most appropriate surface chemical treatments for Sb_2_Se_3_ in substrate configuration, synthesized using a sequential process at very low temperatures (320 °C). Eight different chemical etchings were tested and investigated, and the results show that only KCN-based solutions lead to an improvement in the solar cell’s performance, primarily due to an increase in the fill factor. Surface analysis of the samples shows that KCN etching produces very Sb-rich surfaces that do not affect the properties of the bulk. It is proposed that this Sb-rich interface inverts the surface polarity, creating a “buried junction” with CdS, thereby explaining the improvement of the fill factor of the devices, as confirmed by device modeling. The results of this study underscore the importance of surface management in low-temperature synthesized Sb_2_Se_3_ absorbers, where Sb-rich interfaces are crucial for achieving high-efficiency devices. This research contributes to ongoing efforts to improve the performance of Sb_2_Se_3_ thin film photovoltaic technology and could pave the way for the development of more efficient solar cells with optimized interfaces.

## Introduction

Recent developments in emerging thin film photovoltaic materials based on van der Waals semiconductors demonstrate the unusual structural flexibility of these compounds, which naturally tend to form quasi-one-dimensional (Q1-D) structures.^1^ Being formed by strong covalent bonding in one of the spatial directions, and weaker van der Waals interactions in the other two, their morphology and optic and electric properties can be tuned in a wide range of values by tilting the direction of the Q1-D structures.^2^ To qualify as a van der Waals material, the electronegativity difference between the anion and cation must be lower than 1.5. Several chalcogenides (such as Sb_2_(S,Se)_3_, Bi_2_(S,Se)_3_, GeSe_2_, and SnSe), halides (such as BiI_3_ and SbI_3_), and mixed chalcohalides (such as SbSeI, SbSI, (Sb,Bi)SI, and BiSeI) are currently being investigated as potential candidates.^3−10^

Among all these new materials with exotic properties, Sb_2_Se_3_ has been pointed out as the most promising one until now, demonstrating conversion efficiencies above 10% in a short period of time.^11,12^ This compound consists of covalently bonded (Sb_4_Se_6_)_*n*_ molecules in one of the spatial directions, forming Q1-D ribbons that are held together by nondirectional van der Waals forces in the other two directions, as explained above. These chains of ribbons provide excellent charge transport properties, which render the material an excellent candidate for photovoltaic applications.^13,14^ Moreover, the relatively low synthesis temperatures, that can be as low as 320 °C,^14,15^ lead to properties that are almost unique to Sb_2_Se_3_ over other chalcogenide compounds such as compatibility with thermal sensitive substrates for advanced photovoltaic applications, as well as the possibility of building efficient devices either with a substrate or superstrate configuration^1,13,16,17^

Although Sb_2_Se_3_ has clear advantages over other materials and is composed of only two elements, secondary phases, bulk recombination centers, and interfacial defects are still expected to occur.^18^ According to previously reported simulations, and considering the current state-of-the-art methods, interfacial defects are the most detrimental in terms of the solar cells efficiency degradation, affecting especially the open-circuit voltage (*V*_OC_) of the devices.^1,19^ In this regard, theoretical studies reported by Chen et al. suggest that the introduction of an ultrathin SnO_2_ layer is beneficial for the passivation of the interfacial defects of Sb_2_Se_3_.^20^ This was experimentally confirmed by Mao et al. in superstrate configuration Sb_2_(S,Se)_3_ solar cells. Gon Medaille et al. also demonstrate that either selective surface sulfurization or the introduction of a very thin Al_2_O_3_ layer at the interface could also significantly reduce the interface recombination, boosting the efficiency of the devices.^18^ Experimentally, Feng et al. reported an efficiency enhancement by adding an ultrathin (between 0.1 and 2 nm) Si_3_N_4_ passivating layer between the absorber and the CdS layer in substrate configuration devices.^21^ In addition, Prabhakar et al. observed a passivation effect of selective surface sulfurization, although these materials were investigated for water splitting applications.^22^ With a few exceptions, most of these strategies were applied to superstrate configuration devices using either an intermediate passivating nanolayer or a reactive thermal treatment. Conversely, very few works have been reported about surface passivation in substrate configuration devices and even fewer using chemical etchings.

The importance of surface chemical treatments in substrate configuration thin film solar cells is evident from its relevance in related technologies such as Cu(In,Ga)Se_2_ and kesterite.^23,24^ There have been some attempts to improve the interface with different chemical strategies for the superstrate configuration including the use of ammonium disulfide,^25^ potassium hydroxide,^26^ and carbon disulfide,^27^ among others. Nevertheless, very few attempts have been reported for substrate configuration devices, namely, the use of hydrochloric acid,^28^ which in turn does not improve the conversion efficiency. Table 1 compiles the most relevant chemical etchings published to date for CdTe, CIGS, CZTSSe, and Sb_2_Se_3_, including available device parameters and process conditions. As is clear from this table, limited knowledge on surface treatment of substrate configuration Sb_2_Se_3_ based solar cells is available, considering the long experience accumulated in other relevant thin film chalcogenide technologies. Note then, that the surface treatments under analysis in this study are performed on the surface of antimony selenide exclusively for Mo/Sb_2_Se_3_/CdS/i-ZnO/ITO devices structure and in consequence are valid for devices with substrate configuration. Therefore, this work focuses on the study of different chemical etchings designed for the modification of the Sb_2_Se_3_ surface for substrate configuration solar cells synthesized at very low temperatures (320 °C). For this purpose, Se-rich Sb_2_Se_3_ absorbers were synthesized as previously reported,^15^ which has been identified as a p-type material.^14^ In the first part, different etching solutions, inspired by Table 1, were tested, and solar cell devices were fabricated, in order to select the most promising ones. With the better chemical etching demonstrating an improvement of the solar cell efficiency, a complete analysis of their impact on the fundamental properties of the absorber bulk and surface is presented, in order to understand the best surface treatment for this emerging thin film photovoltaic technology.

**Table 1:** Summary of Most Relevant Etching Procedures Reported for CdTe, CIGS, CZTSSe, and Sb_2_Se_3_ Solar Cell Technologies

TFPV	Etching	Acronym	Description	Surface effects	Device effects
CdTe	HNO_3_-H_3_PO_4_	N-P	CdTe back surfaces and the changes with time of exposure to N-P acid with X-ray photoelectron spectroscopy (XPS), and atomic force microscopy.	As the etching time increases, shifting of tellurium oxide peak toward the higher BE is an indication of increasing contribution from TeO_3_, and the surface roughness increases, as well as the apparent grain sizes.^29^	Formation of a p+ degenerated Te-rich layer. Te-rich layer uniformly covers the CdTe film. So, the Schottky diode formed between the CdTe and metal electrode remains intact, which impairs hole transfer from CdTe to the metal electrode.
	Br_2_-MeOH	Br-M	ARXPS and LEIS-based study of the effect of bromo-methanol etching on commercial Cl-doped p-type CdTe absorbers.	Exponential course of Te enrichment toward the surface.^30^	In this work, they try to improve the back contact between CdTe and Cu/Au. Etching has a strong influence on the stability of the devices but not on the efficiency of the device.^31^
					
CIGS	KCN	KCN	Sputter-deposited CIGS films (in the case of this work, rich in Cu), KCN etching is applied to remove the Cu-Se phases formed on the surface.	It removes excess Cu, going from a Cu-rich surface to a Cu-poor one, avoiding the formation of the Cu-Se secondary phase and leaving a smoother and flatter surface.^23^	Dislocation density and lattice parameter decreased as excess Cu was removed, resulting in an increase in the bandgap and a decrease in the conductivity of CIGS films.
	(NH_4_)_2_S	NHS	(NH_4_)_2_S solution treatment is used as a single way to realize the optimization of the distribution of elements on the CIGS (electrodeposited) surface.	Reduces the surface roughness (better covered by CdS) but also incorporates sulfur (increased bandgap) and removes the impurity phases (reduction of the acceptor Cu-Ga).^32,33^	It reduces series resistance, and consequently increases *V*_oc_, FF, and efficiency.
	Br_2_-MeOH	Br-M	CIGSe layers are prepared by coevaporation and etched in HBr/Br_2_/H_2_O to prepare defined thicknesses.	They find a relationship between the etching time and the final thickness (quasi-constant character of the kinetic), leaving a Se^0^-rich surface, practically constant in composition, over time and eliminating roughness, leaving a flat surface.^34^	Leaves a material with no loss in quality, but with a flat surface that decreases *J*_sc_ and therefore efficiency. Small changes in the bandgap were observed.^35^
					
CZTSSe	KCN	KCN	Effect of KCN on the surface of kesterites (Cu_2_ZnSnS_4_ “CZTS”) by direct and inverse photoemission is investigated. In Cu-poor and Cu-rich condition.	KCN preferentially etches Cu and Sn but not Zn. After KCN etching, they find an increased Eg^Surf^ of 1.91 (±0.15) eV for Cu-poor and 2.45 (±0.15) eV for Cu-rich.^24,36^	The benefit of KCN etching is demonstrated, improving efficiency by 40%, especially *V*_oc_. The changes in the bands create a barrier that prevents recombination at the interface. Thus, improving the band alignment between the kesterite and the CdS.
	(NH_4_)_2_S	NHS	They propose a chemical etching that complements the rest of the etching implemented for kesterite (Cu_2_ZnSn(S,Se)_4_) by removing Sn(S,Se) defects.	Removal of Sn(S,Se), also has a passivation effect on the surface. Depending on the location and size of the defect, it may affect morphology.^37^ The removal of native oxides on the surface is also reported.^38^	Reduces interfacial recombination, increases the quality of the p–n junction, and thus improves the efficiency of devices typically between 20% and 65%.
	Br_2_-MeOH	Br-M	Potential of bromine etching for the study of depth profiles as well as the enhancement of interfaces is demonstrated.	Residual elemental Se is observed on the surface (then removed by combining it with KCN). Concentration of Zn and Ge related defects is reduced.^39,40^	As far as optoelectronic parameters are concerned, the FF improves, and therefore the efficiency (1 point), due to the lowering of the series resistance and the increase in the shunt resistance.
	KMnO_4_-H_2_SO_4_	KMO	They use KMO as etching to remove secondary phases formed in the Zn-rich and Cu-poor conditions.	Removes Zn from the secondary phase ZnSe but leaves a residue of Se_2_, which must be removed by the application of an extra etching.^41^	Substantial improvement in efficiency, improved *J*_sc_ and series resistance, directly related to the removal of ZnSe, and also improvements in *V*_oc_, *R*_sh_, and FF as a result of passivation of surface defects.
					
Sb_2_Se_3_	NH_3_	NH	Sb_2_Se_3_ made by VPD, match the record.	Changed from “clifflike” to “spikelike”.^25^	Improve efficiency by 24%. Reduce carrier recombination, improve band structure, and crystalline orientation.
	KOH	KOH	Sb_2_Se_3_ grown on superstrate by RTE method.	Improve back contact Sb_2_Se_3_/Au. KOH diffuses into the absorber.^26^	Increases the dopant density from 10^13^ to 10^15^ cm^–3^. General rise in optoelectronics (especially *V*_oc_, 70 mV).
	CS_2_	CS	In superstrate configuration with Sb_2_Se_3_ deposited by close space sublimation.	Removes Sb_2_O_3_, but not elemental selenium.^27^	Improves back contact by lowering the barrier potential from 0.43 to 0.26 eV, which lowers the series resistance. No improvement in efficiency due to lower shunt resistance and *J*_sc_.
	(NH_4_)_2_S	NHS	In superstrate configuration with Sb_2_Se_3_ deposited by close space sublimation.	Removes Sb_2_O_3_, but not elemental selenium, and in addition, it increases the amount of metallic selenium.^27^	Improves back contact by lowering the barrier potential from 0.43 to 0.29 eV, which lowers the series resistance. No improvement in efficiency due to lower shunt resistance and *J*_sc_.
	HCl	HCl	Flexible Sb_2_Se_3_ Mo-foil/MoSe_2_/VTD-Sb_2_Se_3_/TE-In_2_S_3_/i-ZnO/ITO	Substantial reduction of Sb_2_O_3_ and selenium metal.^28^	For HCl, there is a decrease in efficiency (from 1.6% to 0.6%). Drastic drop in *J*_sc_, *V*_oc_, and *R*_sh_ parameters. (Other, nonchemical treatments performed on the paper do improve efficiency).

## Experimental Section

Sb_2_Se_3_ thin films were synthesized onto Mo-coated soda–lime glass substrates (SLG/Mo) by a sequential process based on the thermal evaporation of 280 nm of elemental Sb (Sb shots Alfa Aesar, 1–3 mm), followed by reactive thermal annealing under an elemental Se atmosphere. The thermal evaporation of Sb was carried out in an Oerlikon Univex 250 Evaporator, with a base vacuum of 10^–5^ mbar, an evaporation rate of 10 Å/s, and the substrate at room temperature. The reactive annealing was performed in a tubular furnace (Nabertherm RSH 120/750/13) containing a graphite box susceptor with 23 cm^3^ free volume, which additionally contains two crucibles with a total of 25 mg of selenium (12.5 mg each, Alfa Aesar, Se powder 200 mesh 5N). The temperature of the furnace is then increased up to 320 °C at 20 °C/min ramp with a dwell time of 30 min. The samples are cooled naturally to room temperature. The composition of the samples as well as their thicknesses were measured using X-ray fluorescence techniques (Fischerscope X-ray XDAL 237), which was precalibrated using standard samples^15^

Immediately after the synthesis of the absorber, a series of chemical etchings that are summarized in Table 2 were investigated, ensuring that the time between the synthesis of the absorber and the final assembly of the device was as short as possible.

**Table 2:** Summary of Etching Processes under Analysis in the Present Study

		Condition studied			
Etching agent	Acronym	Temp. (°C)	Concentration (% w)	Time	w/wo stirring
HCL	HCl	80	10	[30″, 5′]	w
H_2_SO_4_	KMO	RT	16	[10″, 5′]	w/wo
HNO_3_ -H_3_PO_4_	N–P	RT	0.4 + 29	[5″, 45′′]	w/wo
CS_2_	CS	RT	100	[2′, 60′]	w/wo
KCN	KCN	RT	[2, 10]	[2′, 120′]	wo
(NH_4_)S	NHS	RT	[3, 22]	[1″, 5′]	w
Br_2_ -MeOH	BrM	0	[8, 16]	[4″, 5′]	w/wo
Br_2_-MeOH + KCN	BrM/KCN	0 + RT	8 + 2	2′30′′ + [20′ + 60′]	wo + wo

To complete solar cell devices, n-type CdS was deposited by CBD as the electron transport layer, and finally, i-ZnO and In_2_O_3_(90%)-SnO_2_(10%) (ITO) layers were deposited by DC-pulsed magnetron sputtering (Alliance Concept CT100). This first work was used to select the most promising etching routes for further characterization. Consequently, the insights gained from this research can contribute to the enhancement of the Sb_2_Se_3_/CdS interface in substrate configuration devices.

The Sb_2_Se_3_ layers were characterized before and after the etchings identified as the most promising ones, by using scanning electron microscopy (SEM, Zeiss Auriga Series field emission microscope), with an accelerating voltage of 5 kV and with working distances ranging from 3 to 5 mm.

In addition, X-ray photoemission spectroscopy (XPS) and photothermal deflection spectroscopy (PDS) were used to characterize the reference and as etched samples.

XPS experiments were performed in a SPECS system with a PHOIBOS 150 EP hemispherical energy analyzer with a MCD-9 detector using an X-ray source Al Kα line of 1486.6 eV energy and 200 W power, placed at 54° with respect to the analyzer axis and calibrated by the 3d5/2 line of Ag with a full width at half-maximum (fwhm) of 1.2 eV. The analyzed area was approximately 0.6 μm × 0.5 μm. The selected resolution for the survey (from 0 to 1100 eV of binding energy) was 20 eV of pass energy (PE) and 1 eV/step, while there was 20 eV of PE and 0.1 eV/step for the high-resolution spectra for the main orbitals of the selected elements. The analysis and fitting of the XPS spectra were carried out using the Multipak Version 9.9.08 program from ULVAC-PHI. Measurements are referenced to the C 1s signal, whose binding energy is equal to 284.8 eV in adventitious carbon (from atmospheric contamination). No charge compensation is used in any of these measurements that are made in an ultrahigh vacuum (UHV) chamber, with pressures around 5 × 10^–9^ Torr. The complete set of survey XPS results and the fitting of the Sb 4d orbital for the reference sample are presented in Figure S12 of the SI.

Kelvin-probe force microscopy (KPFM) measurements were performed at room temperature under an inert atmosphere of N_2_ using a Cypher ES Environmental atomic force microscope (AFM) from Oxford Instruments. Measurements were conducted in amplitude modulation (AM-KPFM) with an AC voltage of 1 V at the frequency of the first eigenmode in a two-pass procedure. First, a topographic contour line was recorded in dynamic mode (at a constant amplitude). Second, KPFM was measured, during which the mechanical excitation was switched off, and the tip was made to follow the same contour line, shifted in the *z*-direction by a selected height. Local surface potential (SP) was determined by adjusting the voltage on the probe tip to nullify the amplitude of the oscillatory electrostatic force. For the setup employed, where voltage bias was applied to the tip, a higher (lower) SP corresponds to a lower (higher) surface work function.

The optical absorption of glass (Corning1737)/Sb_2_Se_3_ samples was characterized by PDS. This technique was used to determine the absorptance of the Sb_2_Se_3_ films in the sub-bandgap region. The transverse PDS setup used in this work consists of a 100 W tungsten halogen lamp, a PTI 01-0002 monochromator (two-grating monochromator, spectral range of 400–2000 nm), and a Thorlabs MC1000 optical chopper (4 Hz light modulation frequency). A Signal Recovery 7265 lock-in amplifier was connected to a Hamamatsu C10442-02 PSD position sensitive detector to measure the deflection of a MC6320C 10 mW laser probe beam. Samples were placed in a quartz cell filled with Fluorinert TM FC-40.

The optoelectronic parameters of the complete devices (SLG/Mo/Sb_2_Se_3_/CdS/ZnO/ITO) were characterized through J-V curves carried out with a Sun 3000 AAA-class Abet solar simulator, with a uniform illumination area of 15 cm × 15 cm, calibrated with a Si reference solar cell. The optoelectronic characterization was performed on 3 mm × 3 mm single cells, isolated by mechanical scribing (Micro Diamond MR200 OEG), and without contact grating, nor antireflection coating.

## Results

**Figure 1:**
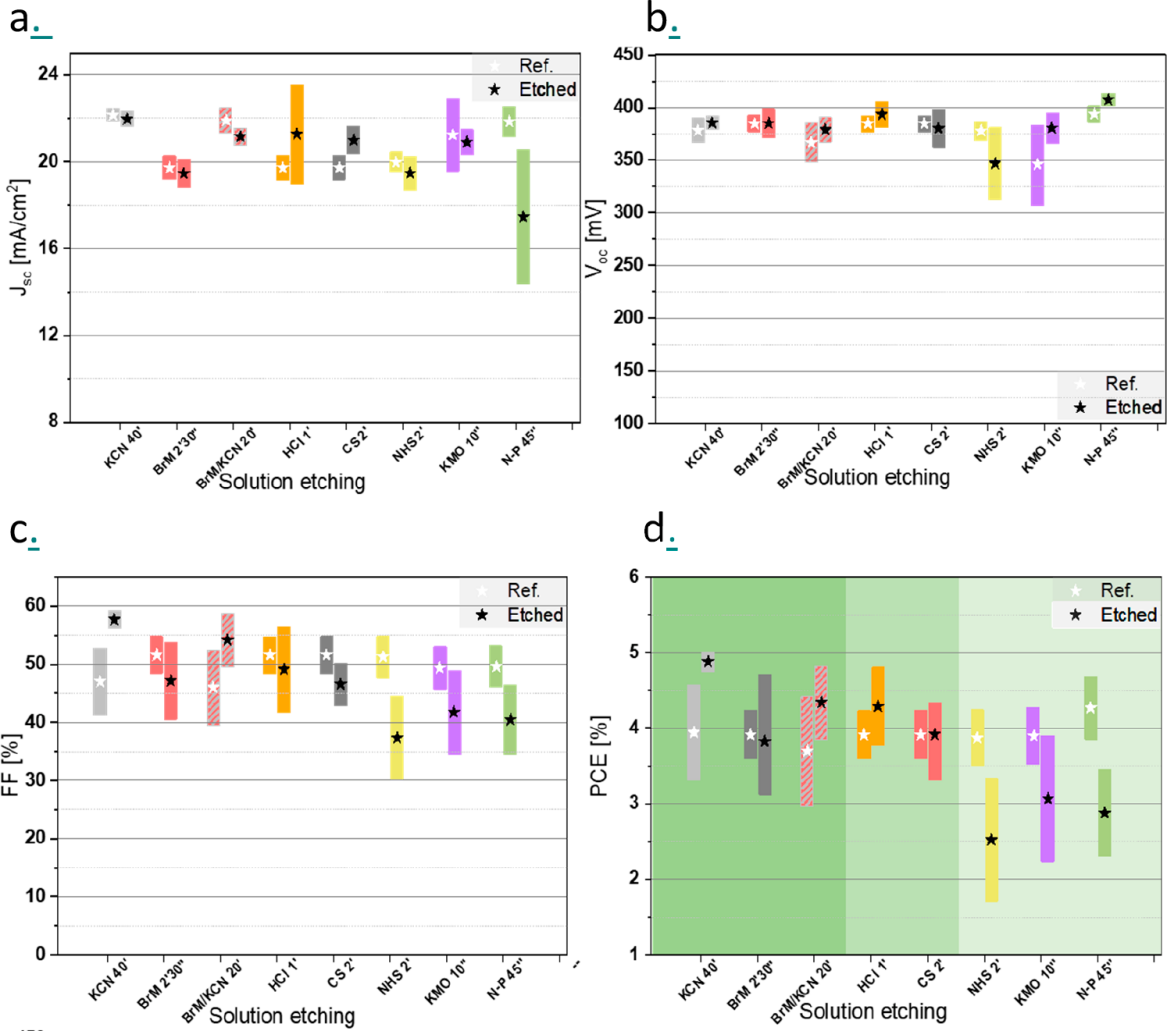
Evolution of the different optoelectronic parameters for solar cells fabricated after applying different chemical etchings. (a) *J*_sc_, (b) *V*_oc_, (c) FF, and (d) PCE. (a) Etchings that improve, have a negligible impact, or deteriorate the PCE are highlighted in dark to light green, respectively.

To begin, it is worth noting that this work focuses on the surface management of Sb_2_Se_3_ absorbers synthesized at unconventionally low temperatures (320 °C). The relevance of the development of low temperature synthetic procedures of Sb_2_Se_3_ is supported by its versatility in terms of device processing, making this material an ideal candidate for temperature-sensitive applications such as tandem solar cells and flexible or transparent substrates. The different etching solutions investigated in this work have been inspired by processes already evaluated and proven effective in other thin film technologies, in particular, HNO_3_-H_3_PO_4_ (NP) and Br_2_-methanol (BrM) in CdTe;^29,42^ KCN, (NH_4_)_2_S (NHS), and BrM in CIGS;^23,32−35^ and KCN, NHS, BrM, KMnO_4_-H_2_SO_4_ (KMO), and CS_2_ in kesterite,^24,36−41^ as presented in Table 1. For all of these chemical solutions, the initial etching conditions were selected from the optimal ones reported in the literature, and a complementary study was performed by varying the etchant concentration and etching times. As a first screening protocol, solar cell devices were fabricated immediately to minimize any possible effects from air exposure. Figure 1 presents the optoelectronic parameters of the best solar cells obtained for each etching process, including the open circuit voltage (*V*_oc_, Figure 1a), the short circuit current (*J*_sc_, Figure 1b), the fill factor (FF, Figure 1c), and the photovoltaic conversion efficiency (PCE, Figure 1d) The averages and the corresponding dispersion values are also reported in the figure. Each experiment was performed by using a reference sample without any etching process before the deposition of CdS as an electron selective contact. In addition, the results obtained for the etching time optimization for all the cases under study are presented in Figures S1–S8 of the Supporting Information (SI).

At first glance, only KCN and to a lesser extent BrM+KCN combo etchants outperform the conversion efficiency of the reference devices (Figure 1d). In particular, KCN exhibits the best results among all the etchings, explained by a large improvement of the FF (Figure 1c), while the *V*_oc_ (Figure 1a) and the *J*_sc_ (Figure 1b) values are almost unaffected. Similarly, in the case of the BrM-KCN combo, most of the increase in the conversion efficiency is due to the improvement of the FF, suggesting that this effect is mainly related to the KCN etching step. Following these two etchings, three other solutions have a negligible or very limited impact on the solar cell optoelectronic parameters, namely, HCl, CS_2_, and BrM. Finally, KMO, N-P, and NHS etchings cause a clear deterioration of the device characteristics, due to a FF decrease in the three cases, together with a markedly lower *J*_sc_ in the case of N-P, and *V*_oc_ in the case of NHS. Due to these findings, HCl, CS_2_, BrM, KMO, N-P, and NHS etchings are discarded for Sb_2_Se_3_ due to their limited or negative impact on the solar cell conversion efficiency.

In summary, KCN etching is identified as the most interesting etching method in terms of device performance, demonstrating an improvement in the FF and conversion efficiency of approximately 20% on average. Of course, one must exercise extreme caution when working with KCN due to its well-established high toxicity, despite the fact that it has consistently demonstrated the most favorable results. The BrM-KCN combo is also identified as promising, as it allows first a reduction in the thickness of the absorber due to the effect of BrM, and then recovery of the efficiency thanks to the positive effect of KCN etching. In the subsequent sections of the article, we exclude the etchings that do not lead to any noticeable or negative changes in the conversion efficiency, with the exception of BrM. The remaining samples, namely, KCN, BrM-KCN, and BrM-etched, follow a comprehensive comparative analysis in comparison to the reference sample. The aim of this analysis is to understand the distinct impact of these etchings on the properties of the Sb_2_Se_3_ absorber and to shed light on the underlying mechanism responsible for the beneficial effect of KCN. Through this analysis, we seek to gain a deeper understanding of the role played by each etching in enhancing the conversion efficiency of Sb_2_Se_3_.

**Figure 2:**
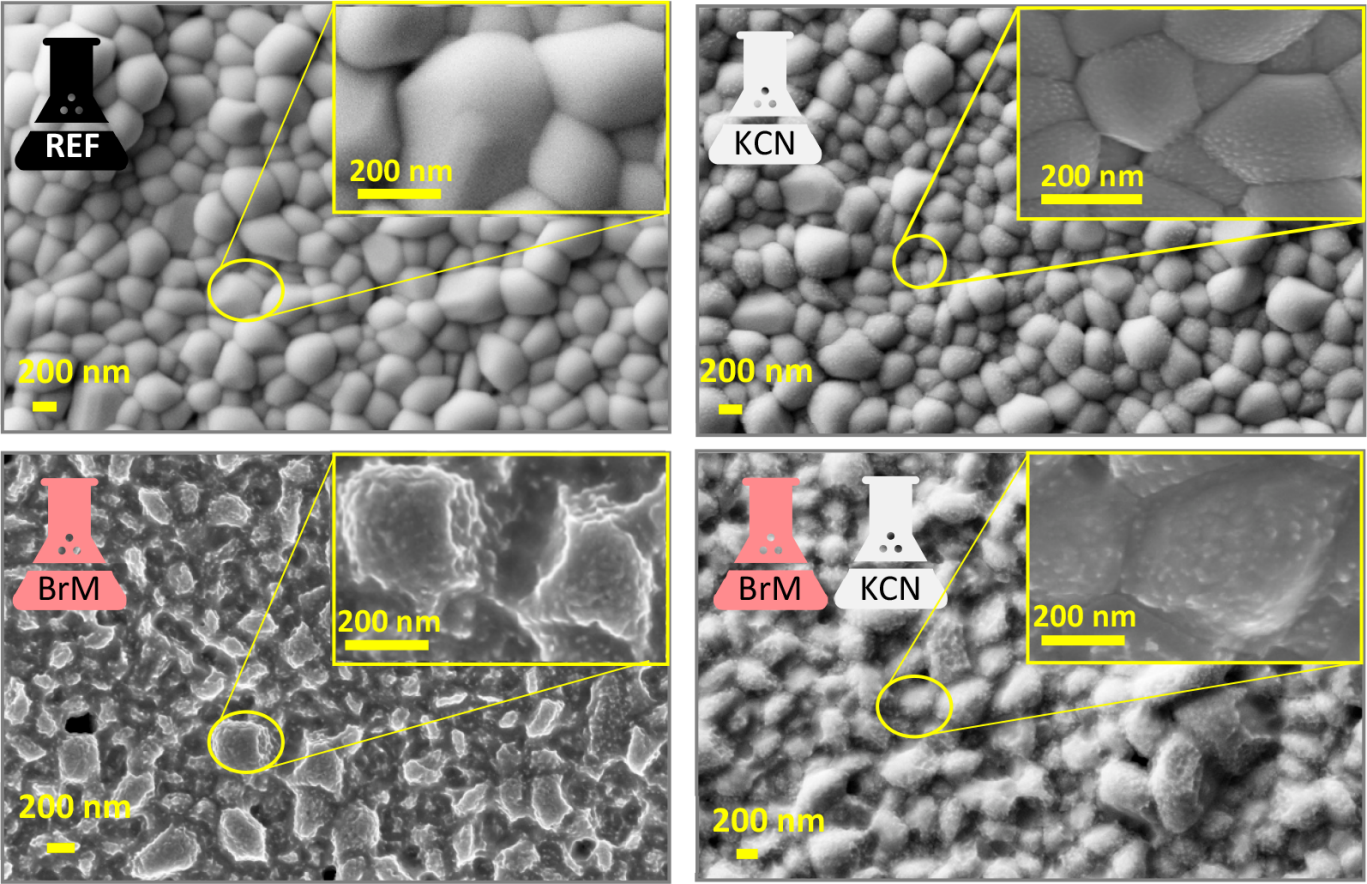
Top view scanning electron microscopy (SEM) images showing the morphology of the as etched samples in comparison with the reference.

Figure 2 shows a detailed surface SEM analysis of the as grown samples as well as those etched with KCN, BrM-KCN, and BrM, together with the reference one. Only one reference sample is shown due to the high process reproducibility. In particular, the as grown Sb_2_Se_3_ layers exhibit large, dense, and compact grains with sizes between 200 and 1000 nm, round in shape, and with a smooth surface. As is evidenced by the SEM Figures, a low density of pinholes is presented in the etched samples. Nevertheless, these pinholes are already presented in the as grown samples (Figure S9 of SI). We believe that due to the low number of pinholes that their possible impact on the optoelectronic properties of the resulting devices can be mitigated by employing i-ZnO at the front contact.

In terms of surface morphology, while KCN only introduces modifications at the grains surface level by the formation of a rougher morphology at the nanoscale, the layers etched with BrM show a drastic change suggesting that Sb_2_Se_3_ is partially dissolved during the etching process. In fact, XRF results shown in Table S1 of the SI confirms that the three etching processes, nominally KCN, BrM, and KCN-BrM combo, reduce the absorber thickness between 20 and 100 nm depending on the etching time. In addition, KCN etching tends to lead to slightly Sb-rich absorbers, while Br-M in principle does not affect the overall composition of the layer. After the dramatic change in the surface morphology upon treatment with BrM solution, the KCN etching is capable of partially recovering the morphology observed in the reference samples. Nevertheless, it is clear that both etching solutions strongly affect the morphology of the layers, and in the case of KCN even the overall layer composition is affected, although this effect is likely more pronounced at the surface level. Despite the reported variations on the surface morphology, Raman spectroscopy analysis performed on the complete devices suggests that CdS is not or only slightly affected by the surface state, as can be observed in Figure S10 of the SI. In particular, the main Raman vibrational mode associated with the S–S vibration of CdS (at 310 cm^–1^ approximately) does not show any significant variation nor in the intensity, neither on the peak position of fwhm.^43^

**Figure 3:**
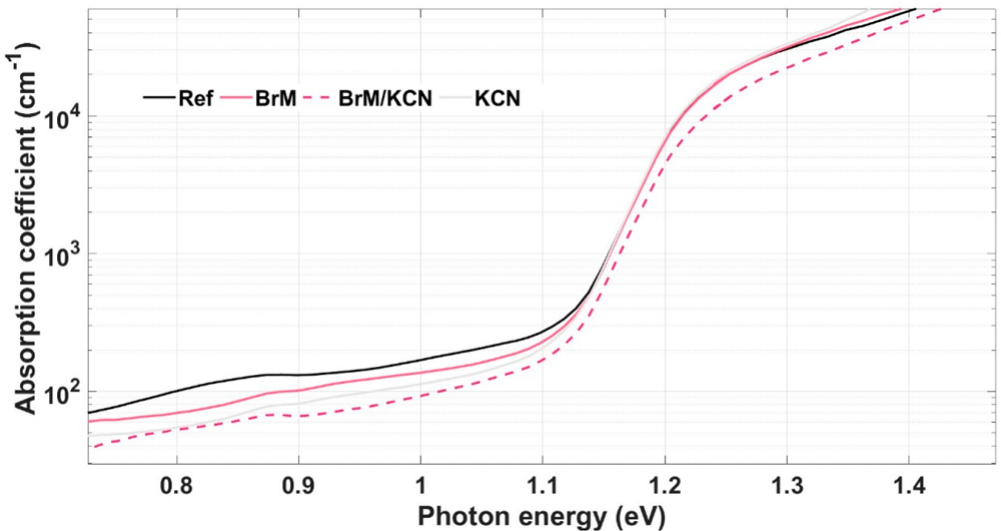
Photodepletion spectra of samples with different surface etchings.

In order to evaluate the extent to which the bulk of Sb_2_Se_3_ is affected by the different etchings, PDS was performed for the selected samples. Figure 3 shows the PDS spectra for the three different etched samples as well as the reference, and Table 3 summarizes the corresponding extracted optical parameters, including direct and indirect bandgaps as well as Urbach energy. The methodology employed to extract all these parameters from the photodepletion spectrum is explained in Figure S11 of the SI. In each case, very similar indirect and direct bandgaps, as well as Urbach energies, are obtained. This suggests that the main effect of all the presented etchings is surface localized, as the etchings do not affect the fundamental optical properties of the Sb_2_Se_3_ bulk nor their quality. Direct bandgap values around 1.20 eV and indirect ones around 1.15 eV are obtained, in agreement with previously published results.^44^ Urbach energies around 23 meV are obtained, which are considered low for this class of solar cells, confirming the low concentration of tail states in these absorbers and that the etchings do not seem to affect the low concentration of these tail states. This value is comparable or even better than results presented for high efficiency chalcogenide solar cells such as those from CIGS and kesterite and are considered very promising from the point of view of bulk quality for Sb_2_Se_3_.^45^ In addition, previous results reported for this material are of the order of those reported in this work.^46,47^

**Table 3:** Most Relevant Optical Parameters Extracted from Fitting of PDS Spectra

Sample	Eg(d) [eV]	Eg(i) [eV]	Eu [meV]
ref	1.20	1.14	23
BrM	1.21	1.14	23
BrM/KCN	1.20	1.17	24
KCN	1.20	1.19	22

The compositional XRF results (Table S1) show that KCN etching resulted in slightly Sb-richer samples, and PDS suggests that these compositional changes happen mainly at the surface of the absorber without affecting the bulk of the material.

**Figure 4:**
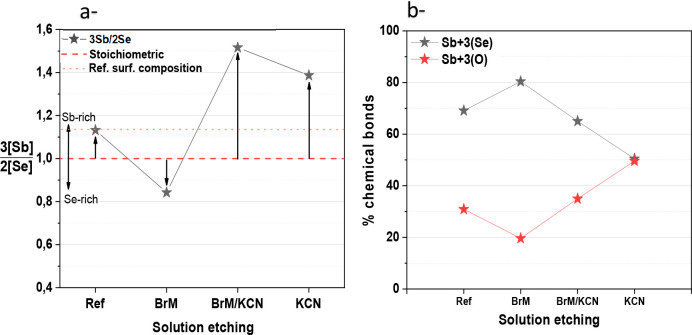
XPS analysis of the different samples (in Figure S12 of the the complete XPS spectra are shown). Evolution of the 3[Sb]/2[Se] ratio with respect to the stoichiometric and reference compositions at the surface (a) and Sb^3+^-Se and Sb^3+^-O relative concentrations (b) for the etchings studied in this work.

To confirm the possible compositional changes at the surface, XPS analysis was performed, focusing on possible changes in the overall Sb and Se concentrations, as well as in the anion bonded to Sb (either Se or O). Figure 4a and b shows the results obtained with the XPS surface analysis for the different etchings (the complete set of XPS results is presented in Figure S12 of the SI). Notably, even if the overall absorber is Se-rich, the surface of the reference samples is slightly Sb-rich with respect to the stoichiometric value (Figure 4a). After the BrM etching, the surface composition changes to slightly Se-rich, becoming in principle with a composition more similar than the bulk composition. This is somewhat expected because, as clearly shown before, BrM tends to remove the surface layer of Sb_2_Se_3_, resulting in a surface that exhibits a composition much more similar to that of the bulk material. On the contrary, if the etching includes a KCN etching step, the composition changes toward a very Sb-rich surface, as is clear in the two cases under study, independently if the KCN was applied directly on the reference sample (slightly Sb-rich surface) or in the BrM etched sample (slightly Se-rich surface). This confirms that KCN transforms into a surface composition that is rich in Sb, independent of the starting condition. It is well-known that Sb-rich Sb_2_Se_3_ exhibits n-type polarity,^48^ strongly supporting the formation of an inverted polarity region at the surface. This way, and close to the surface, a p-type-Sb_2_Se_3_/n-type-Sb_2_Se_3_ quasi-homojunction is formed, inducing a type of “buried junction” similar to what is reported on CIGS when heavy alkali post deposition treatment is applied.^49^ The space charge region is then shifted from the defect-prone Sb_2_Se_3_/CdS interface where the lattice mismatch between both materials often leads to recombination, toward a less defective configuration p-type-Sb_2_Se_3_/n-type-Sb_2_Se_3_ interface. This polarity inversion of the Sb_2_Se_3_ surface after KCN etching can explain the observed conversion efficiency improvement in the solar cell devices in Figure 1d, especially the improvement on the FF.

On the other hand, the evolution of the Sb^3+^ bonded to Se and O is shown in Figure 4b. As shown in the inset of Figure S12, Sb 4d doublet peaks bound to Se are located at 33 and 34.2 eV for 4d^5/2^ and 4d^3/2^ orbitals, respectively, while Sb 4d peaks bound to O are located at 34.7 and 35.9 eV. For the absorber etched with BrM, the increase in Se concentration at the surface leads also to an increase in the concentration of Sb^3+^ bound to Se. For the etchings involving KCN, and as expected, the concentration of Sb^3+^ bound to O is increased due to the higher availability of Sb at the surface, which can rapidly oxidize when in contact with the atmosphere. This Sb_2_O_3_ can be partially dissolved during the CdS electron transport layer deposition while still keeping an overall Sb-rich layer at the surface. Experimental validation is required to corroborate this hypothesis.

The corresponding reactions happening for the two etchings follow the previous knowledge from other chalcogenide technologies (CdTe, CIGS, CZTS), and the effect of both etchants can be summarized as follows:

Br_2_-MeOH: This chemical agent reacts with the chalcogen oxidizing it from (−2) to (0) oxidation state, whiles Br_2_ is reduced to (−1) oxidation state through the following reaction:
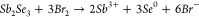


KCN: There is no general consensus about the reactions associated with KCN etching in chalcogenides, but in general it is accepted that KCN efficiently dissolves Se^0^ in basic media through the following reaction:
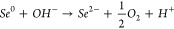


In our case, the pH of the KCN solution is very basic (pH 13.5), suggesting the high probability of the occurrence of this reaction, converting the surface to Sb-rich, as confirmed by XPS measurements in Figure 4.

In the case of the combined etching using first Br_2_-MeOh and then KCN, the elemental selenium created by the first solution is then dissolved by the second, in full agreement with XPS measurements.

We lack direct evidence that these chemical solutions do not infiltrate the absorber; theoretically, they should not, but the possibility of entry through pinholes or grain boundaries cannot be entirely dismissed. However, based on the data collected thus far, we can confidently assert that KCN has had no adverse effects on the absorber, either at the surface or within the bulk. It has consistently demonstrated improvements in efficiency rather than declines. To validate these hypotheses, measuring the thickness of the SbO_3_ layer and conducting depth profile analyses without impacting the surface would be advantageous.

**Figure 5:**
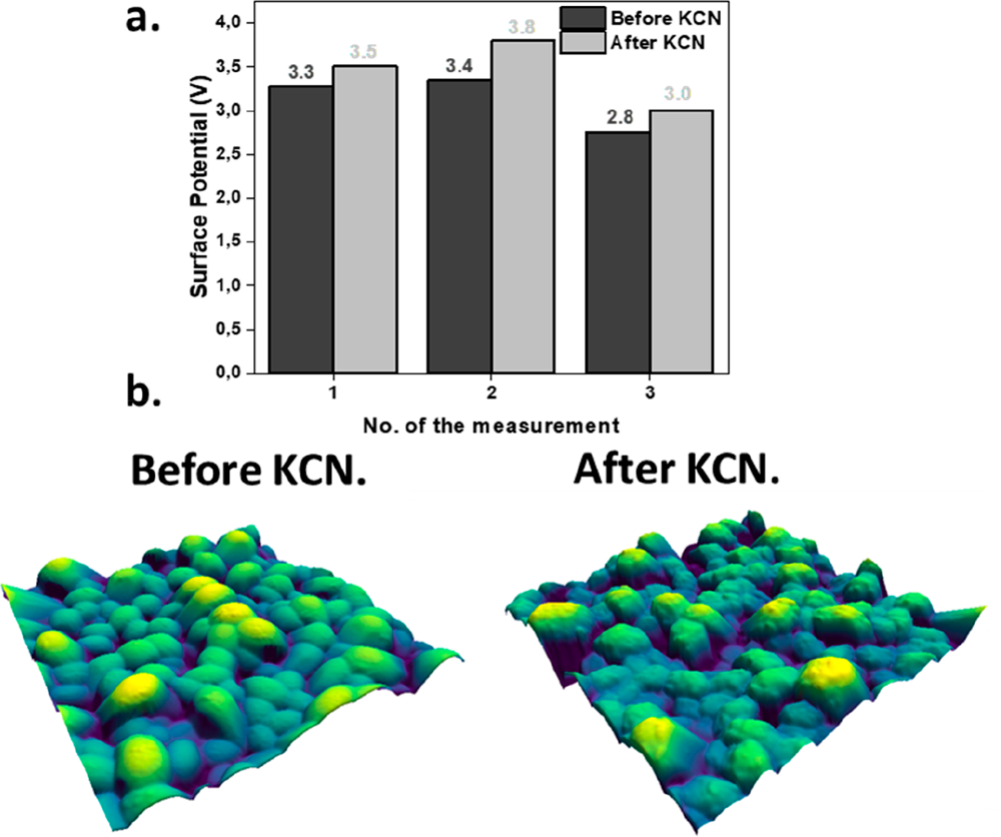
(a) Values of the surface potential obtained by KPFM for the sample before and after KCN etching. (b) Three-dimensional visualization of the merged topographic and surface potential data before (left) and after (right) the etching.

To observe the effect of KCN on the band alignment, a comparative study was performed by KPFM for the same surface absorber with and without KCN etching (Figure 5a and b). Three measurements were performed with the same tip on both surfaces to evaluate the changes. As seen in Figure 5a, the surface potential (SP) is larger for the films etched with KCN, indicating a decrease in the work function. The decrease of the work function confirms the “buried-junction” hypothesis. This observation is consistent with a surface doping from p- to n-type, leading to the formation of a shallow buried junction. Hence, carrier separation occurs within the Sb_2_Se_3_ absorber rather than at the defective interface with the CdS. This permits it to mitigate their influence and consequently decreases the series resistance of the device, while increasing its FF. The topographical images also clearly indicate an increase in roughness in the surface of the grains produced by KCN etching as observed by SEM (Figure 2).

**Figure 6:**
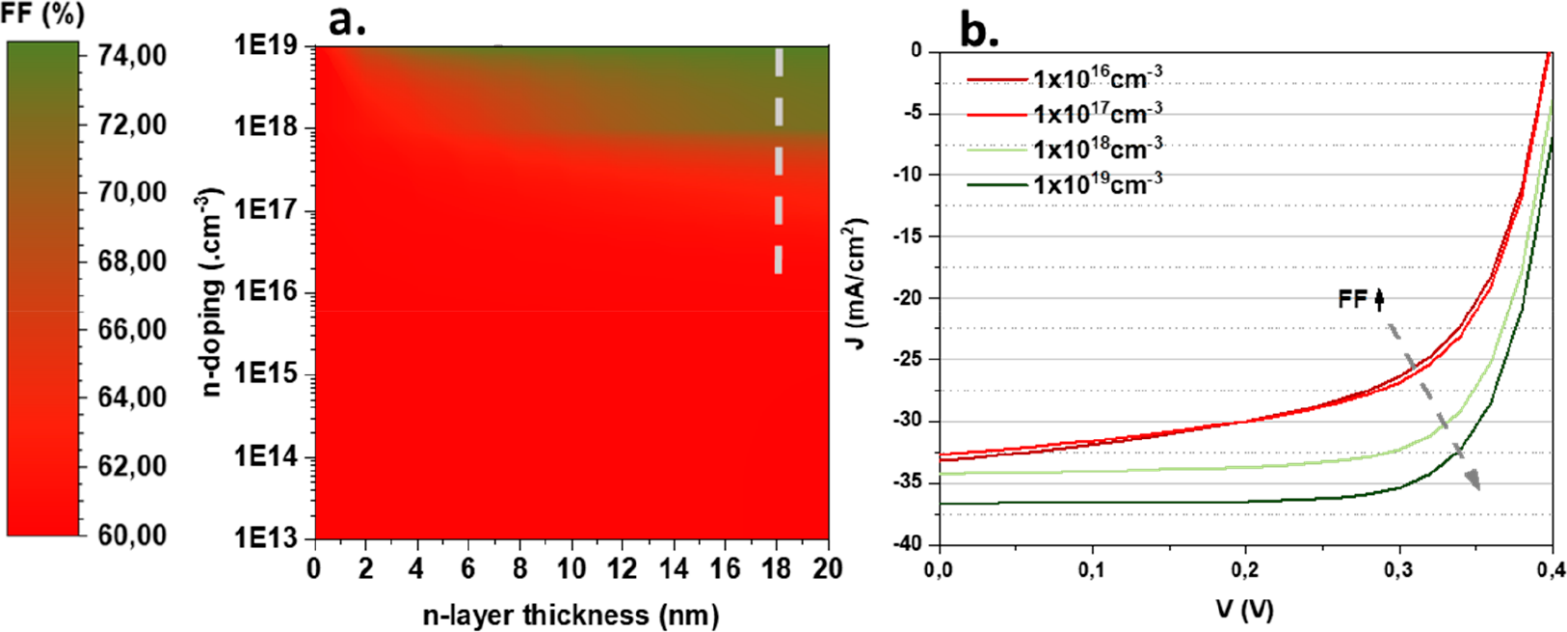
Surface plot of the modeled evolution of the FF as a function of the surface n-doping depth and doping level (a). Corresponding J-V curves (b). The blue arrow illustrates how the FF is improved by the increase in the surface doping level.

To test the hypothesis of a buried homojunction following KCN etching, we conducted numerical simulations investigating the influence of an ultrathin n-doped layer on the surface of the absorber in a SCAPS-1D model, building upon the previously reported baseline that simulates the behavior of Sb_2_Se_3_.^18^ We varied the doping level of the n-type surface layer from 10^13^ to 10^19^ cm^–3^ and the doping depth from 2 to 20 nm, while keeping other parameters constant, including the interface defect properties. As the exact characteristics of this doping layer are currently unknown, we performed a parametric variation of both doping level and depth simultaneously to create surface plots for each photovoltaic figure of merit. While all four figures of merit were calculated, we report only on the fill factor here (Figure 6a) with the complete data set in the Supporting Information (see Figure S13 of the SI). The corresponding J-V curves directly from SCAPS along with a trend arrow are shown in Figure 6b for illustrative purpose. Our findings suggest that while *V*_oc_ and *J*_sc_ show limited variations (though a visible improvement of the latter is observed in selected cases), the FF is significantly improved in instances of n-doping above 10^17^ cm^–3^, from 60% for the reference case without n-doping up to 74% for an edge case of 10^19^ cm^–3^ doping. The qualitative agreement with the specific increase in FF obtained in the experimental results lends support to our hypothesis that KCN etching facilitates the creation of a buried junction. We acknowledge that our model cannot be considered quantitative, but it does also suggest that surface n-doping must reach a certain threshold to achieve an improvement in FF (here, 10^17^ cm^–3^ and above), as observed in the experimental results. This numerical simulation supports our hypothesis that a Sb-rich Sb_2_Se_3_ surface can invert the surface polarity leading to a buried junction structure, largely improving the FF of Sb_2_Se_3_-based solar cells.

**Figure 7:**
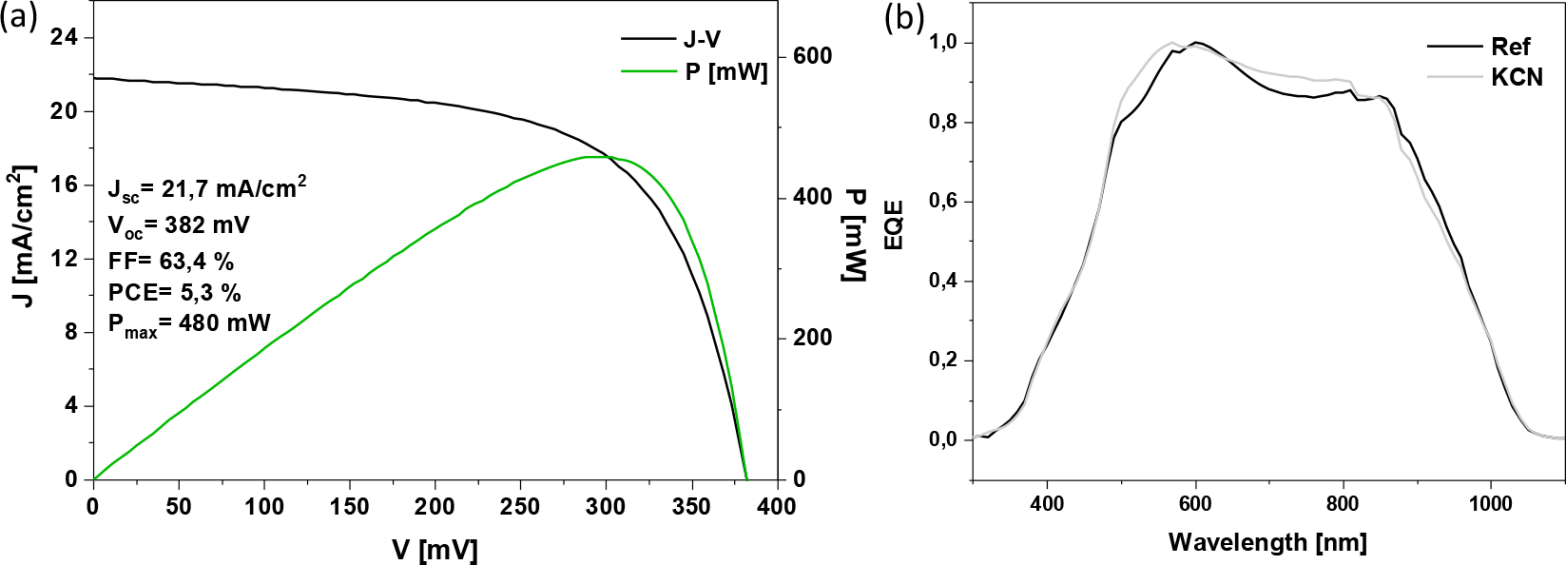
AM1.5 illuminated J-V curve with the corresponding optoelectronic parameters for optimized devices etched with KCN before the deposition of CdS as an electron selective contact (a). EQE spectra of reference and KCN etched devices (b).

Figure 7 shows the J-V AM1.5 illuminated curve with the corresponding optoelectronic parameters, and the corresponding external quantum efficiency (EQE) for an optimized solar cell device, with a PCE of 5.3%, among the highest reported for Sb_2_Se_3_ deposited by a sequential process and substrate configuration devices.^50−52^ The EQE spectrum shows an improved collection between 500 and 600 nm of the KCN etched device with respect to the reference, which is below the CdS absorption threshold, thus supporting the idea of an improved p–n interface consistent with the buried-junction hypothesis. An improvement in quantum efficiency at high energies is often due to a reduction in p–n interface recombination, where both holes and electrons are generated close to the defective interface. On the other hand, lower energy photoelectrons being generated deeper in the absorber are in comparison less affected by those interface defects as no photoholes are present in the space charge region, thus leading to a recombination bottleneck. In summary, we demonstrate the importance of generating Sb-rich surfaces for efficient Sb_2_Se_3_ solar cells using substrate configuration and CdS as an electron selective contact. The use of KCN appears as a simple pathway to obtain such Sb-rich surfaces, independent of its previous history and/or composition.

## Conclusions

In conclusion, the selective surface treatment of the Sb_2_Se_3_ photovoltaic absorber using different etching solutions, inspired by conventional chalcogenide technologies, has been demonstrated to be an effective method to enhance the performance of solar cell devices. Our investigation reveals that KCN and KCN combined with Br-MeOH etchings are the most effective methods, while other etchings have a negligible or even negative impact on device performance.

The fundamental optical properties of the Sb_2_Se_3_ absorber remain unaffected by the different etchings, as demonstrated by PDS analysis. However, a compositional analysis of the surface reveals that KCN etching results in a highly Sb-rich surface, regardless of its previous composition. This inversion of the surface polarity forms a “buried junction”, which explains the improvement of the fill factor and, consequently, the power conversion efficiency. Our device simulations further support that the interplay between p-type and n-type Sb_2_Se_3_ holds high promise for improving the efficiency of this type of solar cell in the future.

Overall, this study provides valuable insights into the surface treatment of Sb_2_Se_3_ absorbers and their impact on the performance of solar cells, paving the way for the development of even more efficient and cost-effective solar energy conversion technologies.

## Supporting Information Available

Evolution of optoelectronic parameters corresponding to the various etching procedures conducted. Evolution of the absorber’s thickness and chemical composition, as depicted in the XRF data. Model employed for calculating Urbach energies, along with the XPS survey results, as well as all the graphs extracted from our simulations (PDF)
