# Supplementary material for: KCN Chemical Etching of van der Waals Sb2Se3 Thin Films Synthesized at Low Temperature Leads to Inverted Surface Polarity and Improved Solar Cell Efficiency
Source: ACS Appl Energy Mater. 2024 Jan 22;7(3):874–84. doi: 10.1021/acsaem.3c01584 (PMC10865286; doi:10.1021/acsaem.3c01584)
Supplement: Supplementary file 1 — ae3c01584_si_001.pdf [file ae3c01584_si_001.pdf]

Supporting information.

KCN chemical etching of van der Waals Sb_2Se_3 thin films synthesized at low temperature leads to inverted surface polarity and improved solar cell efficiency

*Maykel Jiménez-Guerra[1,2], Lorenzo Calvo-Barrio[3,4], Jose Miguel Asensi[5], Ivan Caño-Prades[1,2], Shunya Yan[6], Esther Barrena[6], Joaquim Puigdollers[1,2], Zacharie Jehl[1,2], Yudania Sánchez[7], Edgardo Saucedo[1,2]

Affiliations

[1]- Universitat Politècnica de Catalunya (UPC), Photovoltaic Lab – Micro and Nano Technologies Group (MNT), Electronic Engineering Department, EEBE, Av Eduard Maristany 10-14, Barcelona 08019, Catalonia, Spain.

[2]- Universitat Politècnica de Catalunya (UPC), Barcelona Center for Multiscale Science & Engineering, Av Eduard Maristany 10-14, Barcelona 08019, Catalonia, Spain.

[3] Centres Científics i Tecnològics (CCiTUB), Universitat de Barcelona, C. Lluis Solé i Sabaris 1-3, 08028 Barcelona, Spain

[4] IN2UB, Departament d'Enginyeria Electrònica i Biomèdica, Universitat de Barcelona, C. Martí i Franquès, 1, 08028 Barcelona, Spain

[5] Departament de Física Aplicada, Universitat de Barcelona, C. Martí i Franquès, 1, 08028 Barcelona, Spain

[6] Institut de Ciència de Materials de Barcelona (ICMAB), Carrer dels Til·lers, Bellaterra 08193, Spain

[7] Institut de Recerca en Energia de Catalunya (IREC), Jardins de les Dones de Negre, 1, 08930 Sant Adrià del Besòs, Spain

Corresponding author.

Maykel Jiménez Guerra (Maykel.jimenez@upc.edu)

Permanent address: Universitat Politècnica de Catalunya, Av. Eduard Maristany, 16, 08019 Barcelona, Spain

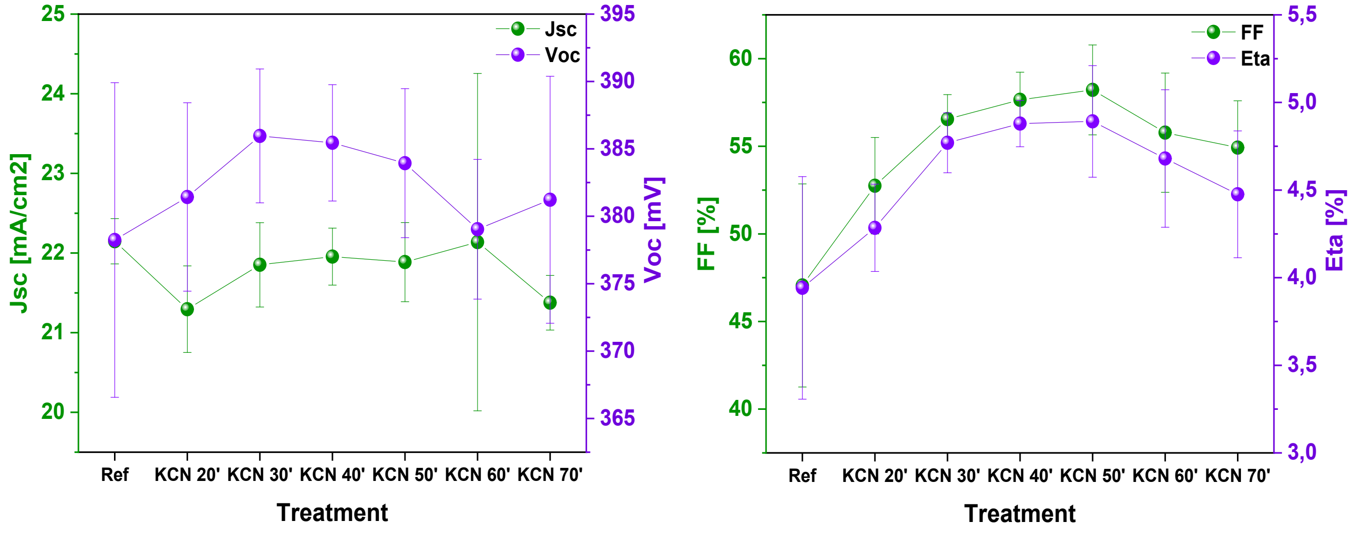

Figure S1. Evolution of the different optoelectronic parameters for solar cells fabricated after applying different times for a KCN treatment (2%). (a) Jsc and Voc, (b) F.F. and PCE.

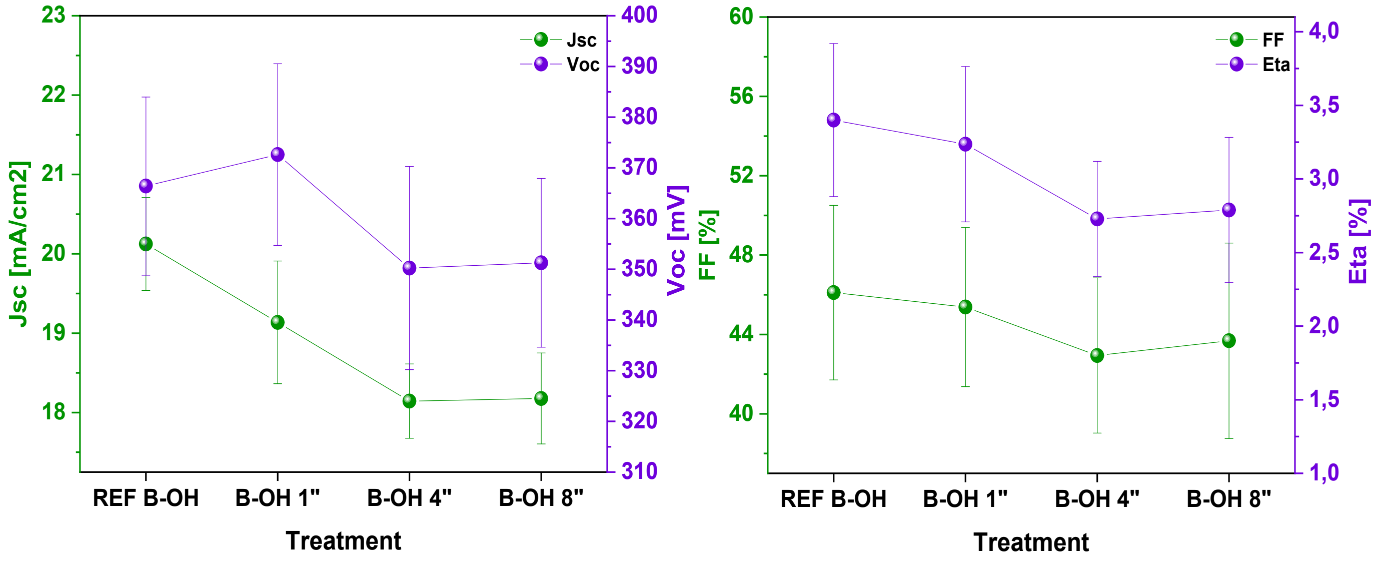

Figure S2. Evolution of the different optoelectronic parameters for solar cells fabricated after applying different times for a BrM treatment (8%). (a) Jsc and Voc, (b) F.F. and PCE.

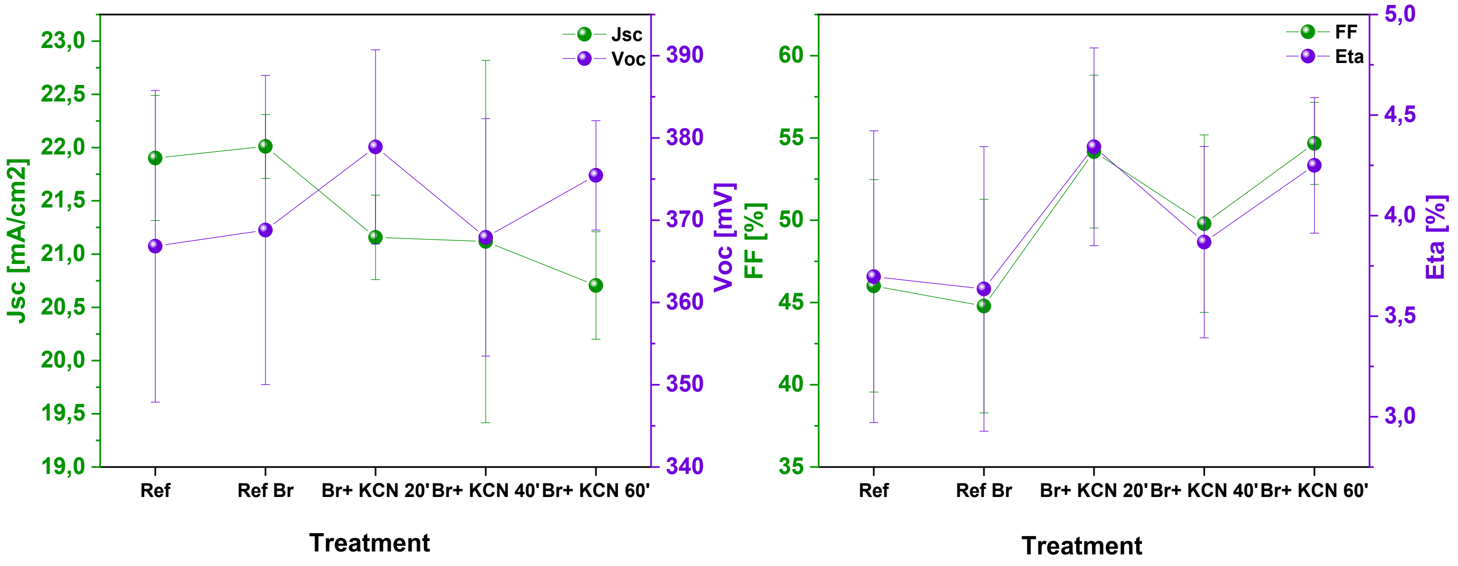

Figure S3. Evolution of the different optoelectronic parameters for solar cells fabricated after applying different times for a BrM/KCN treatment (8%/2%). (a) Jsc and Voc, (b) F.F. and PCE.

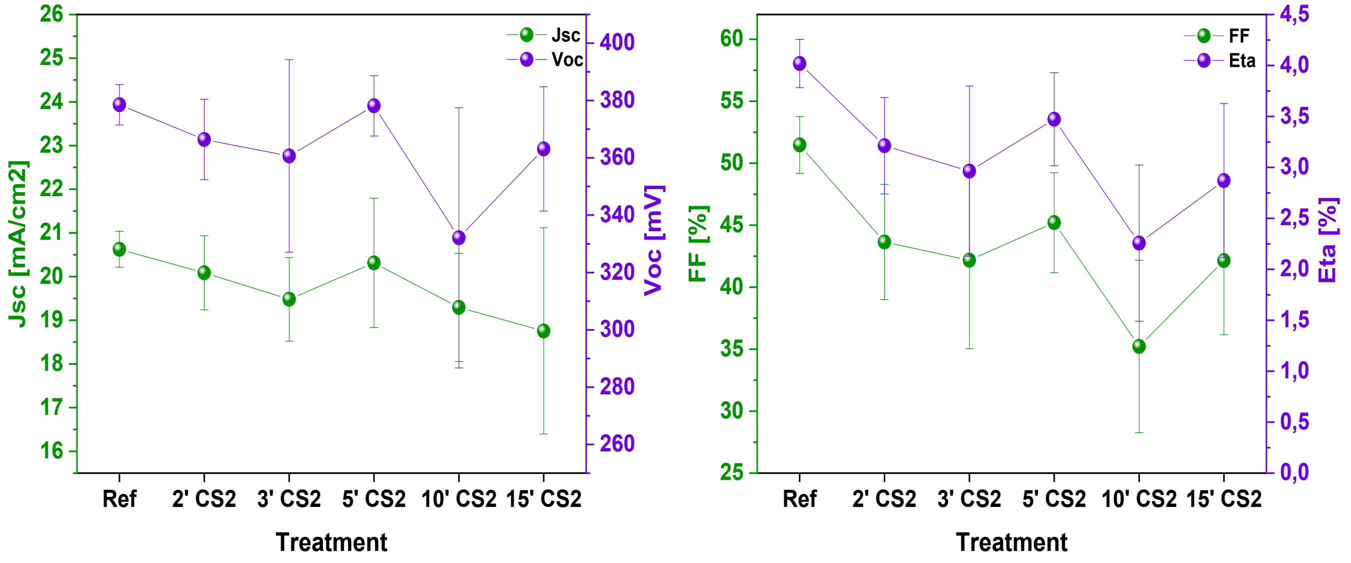

Figure S4. Evolution of the different optoelectronic parameters for solar cells fabricated after applying different times for a CS treatment (pure). (a) Jsc and Voc, (b) F.F. and PCE.

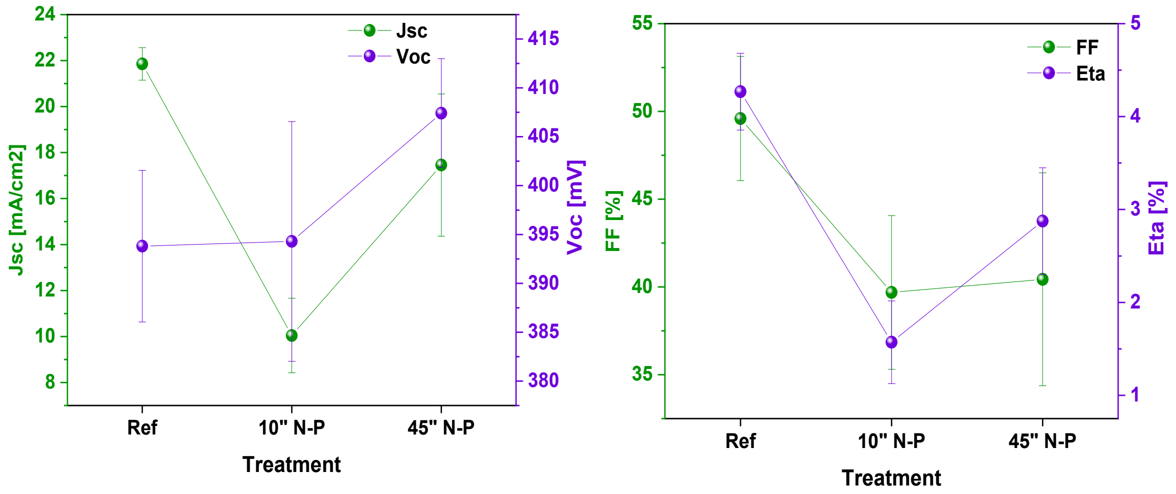

Figure S5. Evolution of the different optoelectronic parameters for solar cells fabricated after applying different times for a N-P treatment (0,4% 29%). (a) Jsc and Voc, (b) F.F. and PCE.

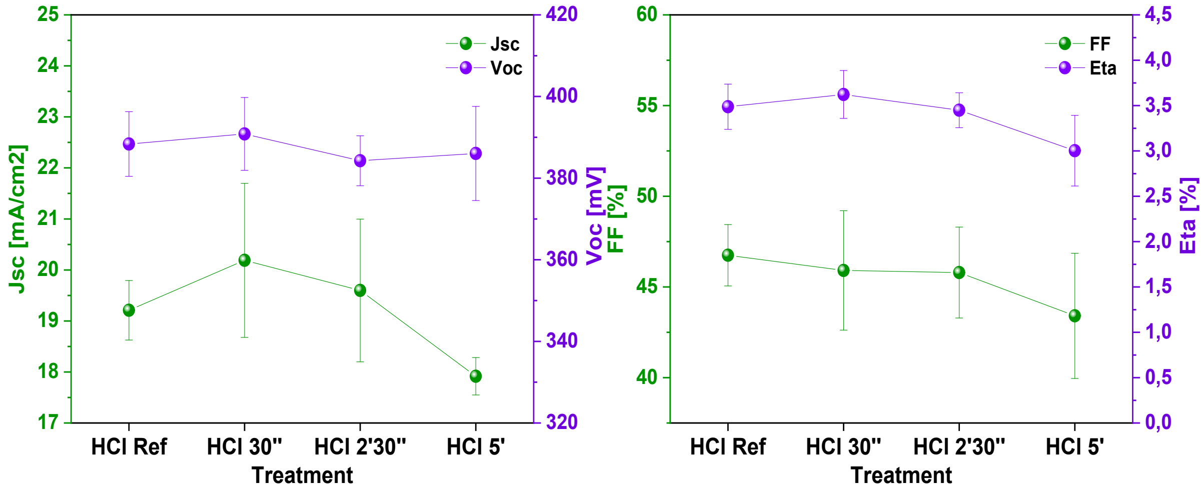

Figure S6. Evolution of the different optoelectronic parameters for solar cells fabricated after applying different times for a HCl treatment (10%). (a) Jsc and Voc, (b) F.F. and PCE.

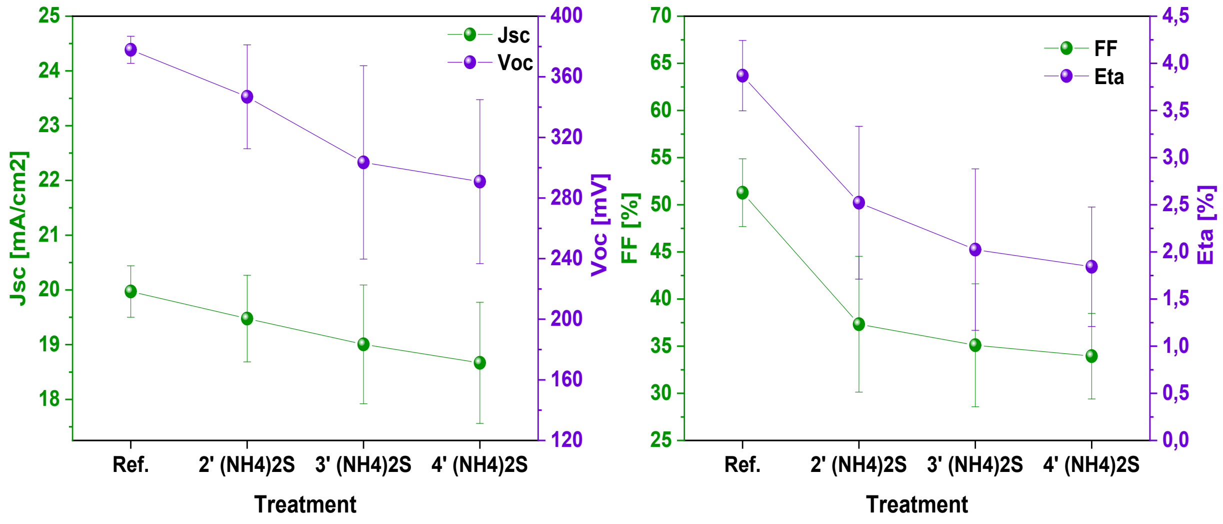

Figure S7. Evolution of the different optoelectronic parameters for solar cells fabricated after applying different times for a NHS treatment (2%). (a) Jsc and Voc, (b) F.F. and PCE.

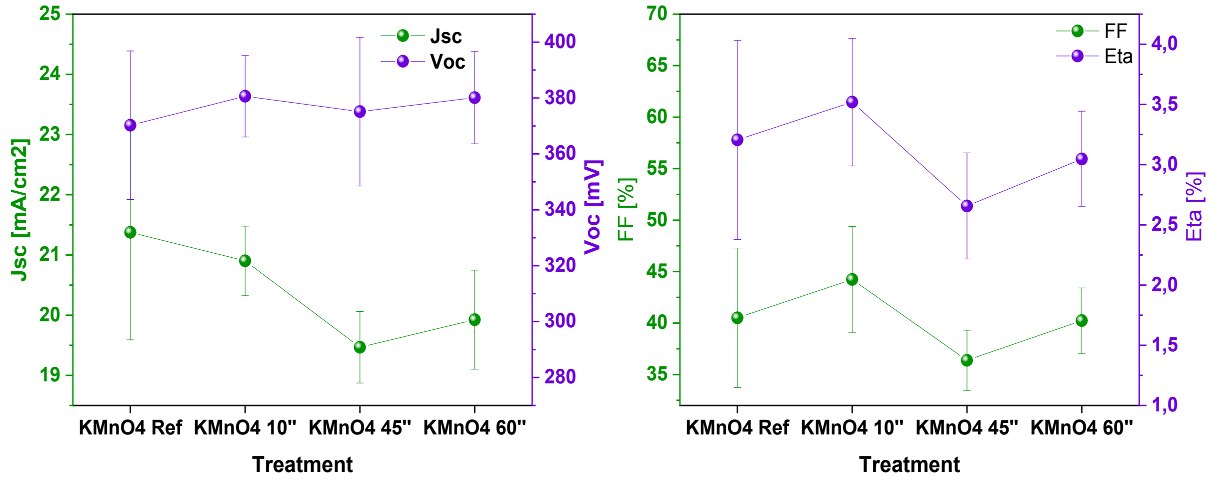

Figure S8. Evolution of the different optoelectronic parameters for solar cells fabricated after applying different times for a KMO treatment (16%). (a) Jsc and Voc, (b) F.F. and PCE.

Table S1. Evolution of thickness and absorber composition by XRF

	Time	Before etching			After etching		
		Thickness (nm)	Sb (%)	Se (%)	Thickness (nm)	Sb (%)	Se (%)
KCN	REF	557±4	37.08±0.3	62.92±0.3	-	-	-
	20’	583±2	37.17±0.3	62.83±0.3	551±3	37.21±0.5	62.79±0.5
	60’	569±7	37.34±0.3	62.66±0.3	526±6	37.39±0.5	62.61±0.5
	90’	558±8	37.16±0.3	62.84±0.3	485±9	37.39±0.3	62.66±0.3
Br-M	Ref	529±14	37.29±0.4	63.71±0.4	-	-	-
	2’	552±10	37.15±0.22	62.85±0.22	430±15	37.26±0.3	62.75±0.3
	2’30”	561±9	37.37±0.3	62.63±0.3	440±13	37.31±0.4	62.69±0.4
	3’	538±12	37.24±0.3	62.76±0.3	441±12	36.88±0.7	63.12±0.7

Time	Before etching			After first etching			After second etching		
	Thickness (nm)	Sb (%)	Se (%)	Thickness (nm)	Sb (%)	Se (%)	Thickness (nm)	Sb (%)	Se (%)
2’30’+20’	764±12	35,75±0,21	64,25±0,21	743±12	35,45±0,08	64,55±0.08	710±16	36,61±0,21	63,39±0,21

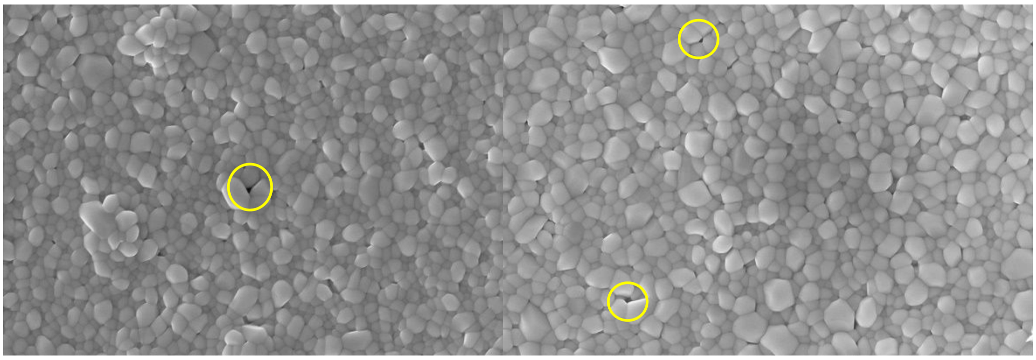

Figure S9. Additional SEM images of the reference reveal the presence of pinholes, marked with yellow circles some of them.

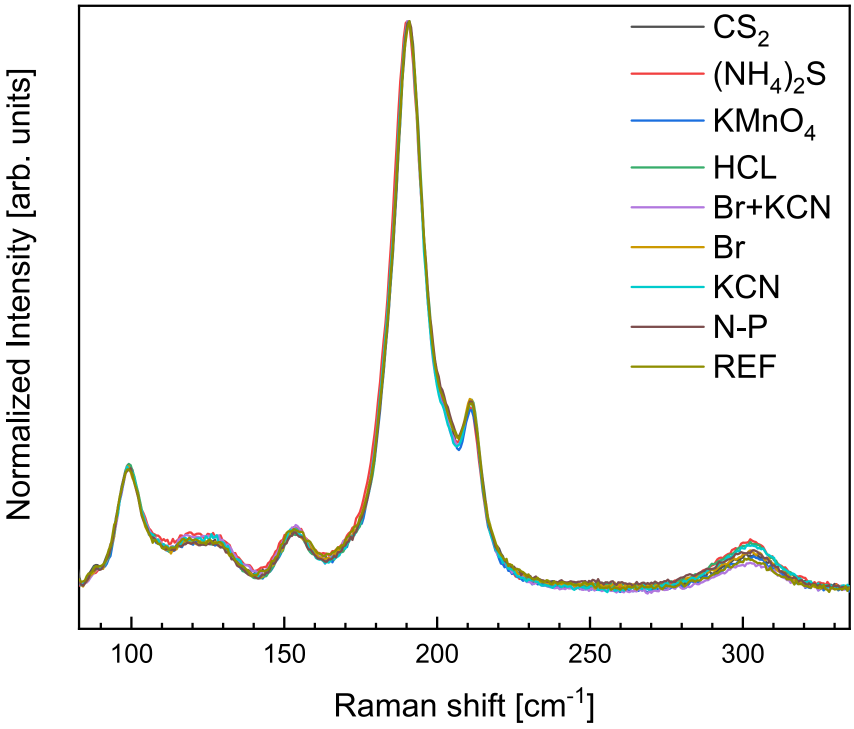

Figure S10. Presentation of the Raman measurements of all the etching carried out in the study.

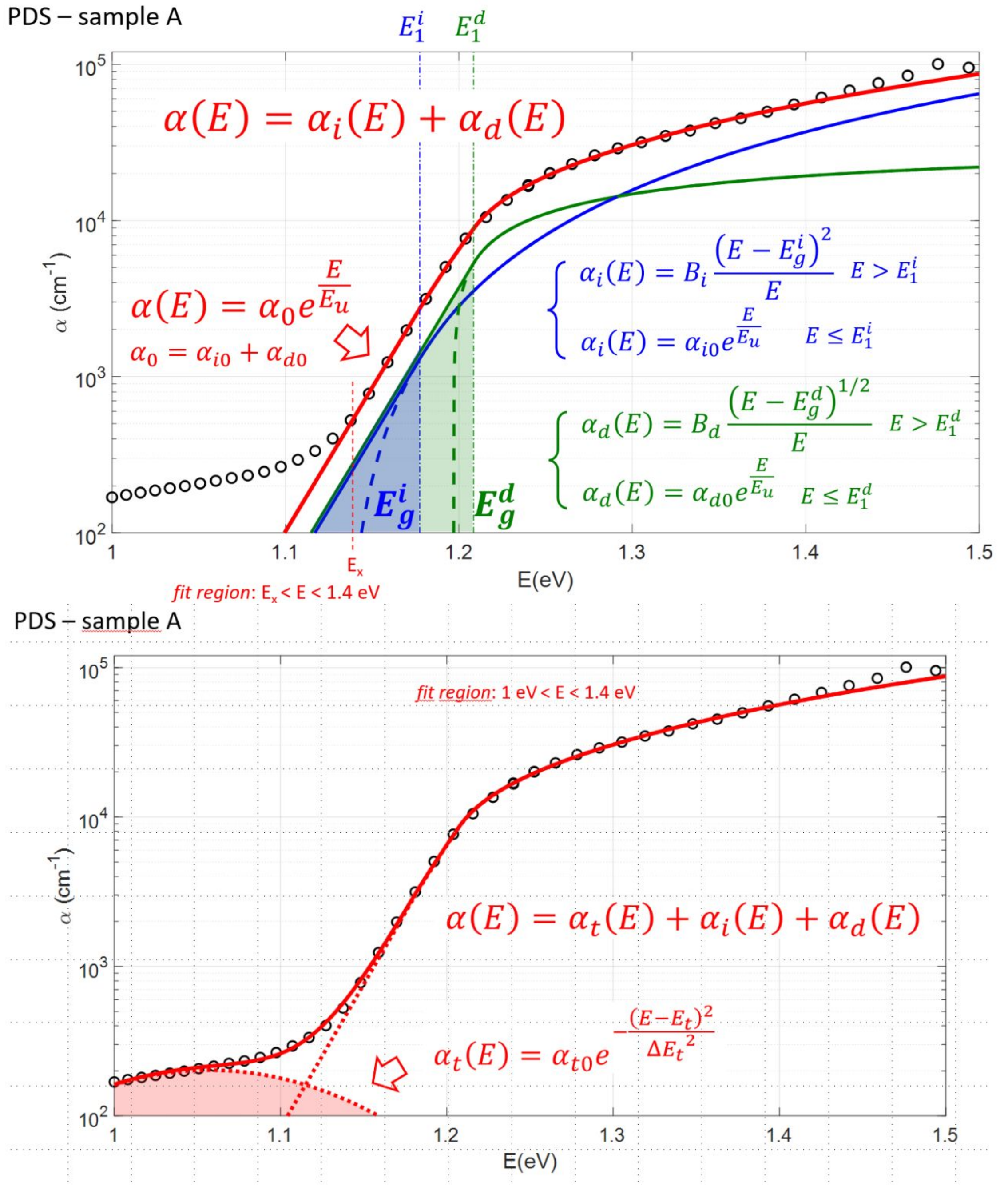

Figure S11. Presentation of the model used with two gaps, one direct and one indirect, which fits perfectly with the experimental data. To estimate the defect level of the material, a Gaussian is used to reproduce the absorption by localized defect states, with the prefactor of the Gaussian serving as a measure of the defect level. The simplicity of the Gaussian fit helps to correct the value of the Urbach energy, as the effect of subgap absorption tends to increase the value of Eu. [53]

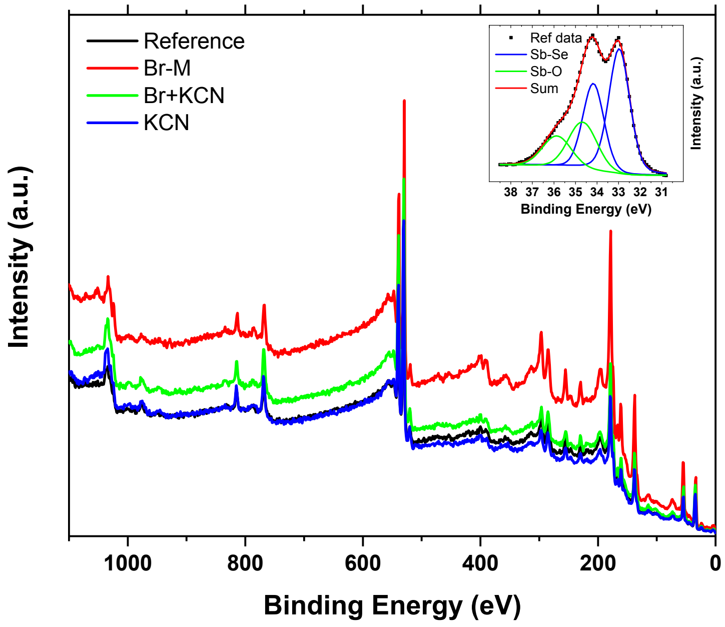

Figure S12. Complete set of XPS results

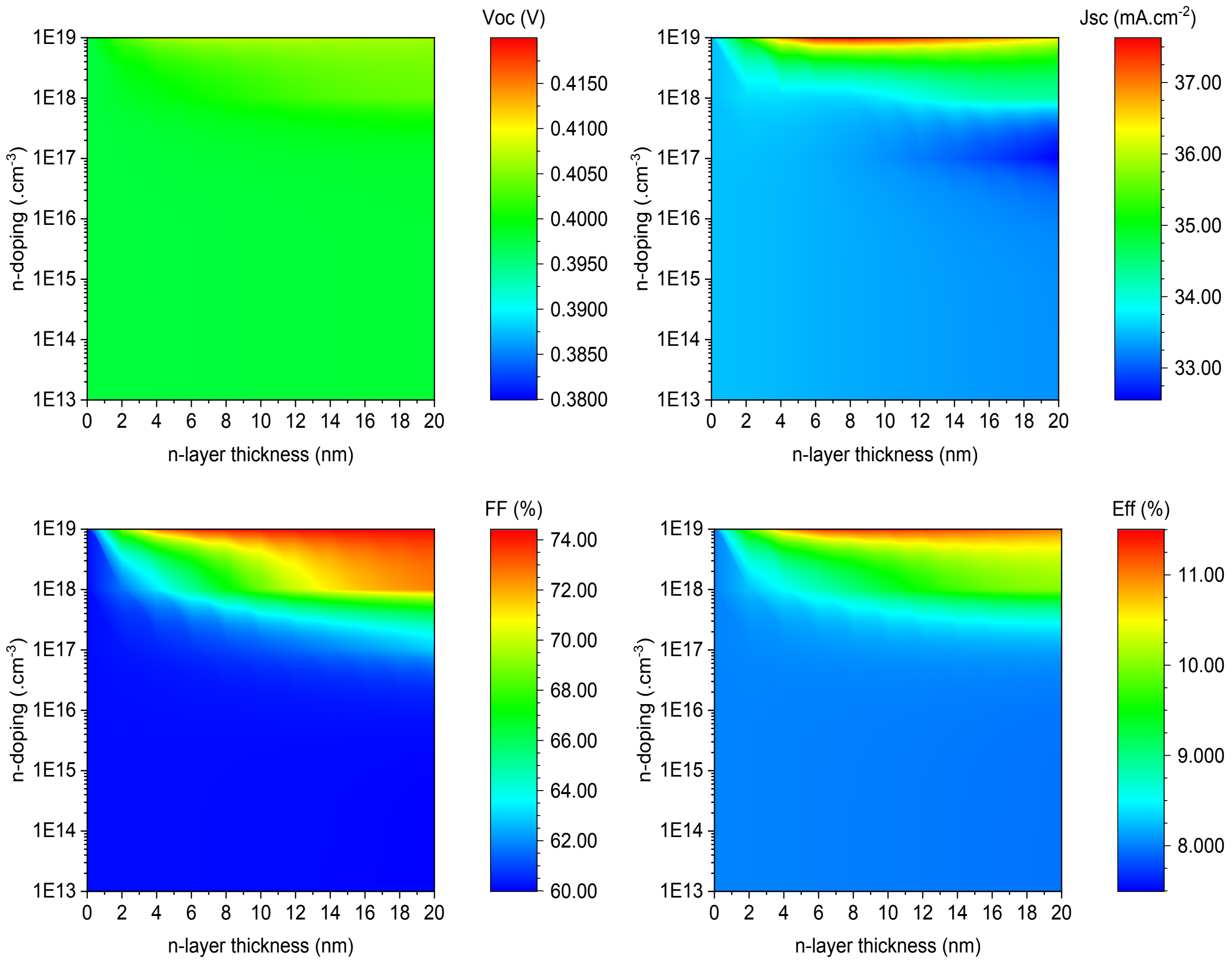

Figure S13. Simulation
